# Curing Opioid Toxicity with Intrathecal Targeted Drug Delivery

**DOI:** 10.1155/2019/3428576

**Published:** 2019-05-16

**Authors:** Thomas P. Pittelkow, Markus A. Bendel, Jacob J. Strand, Susan M. Moeschler

**Affiliations:** ^1^Division of Pain Medicine, Department of Anesthesiology and Perioperative Medicine, Mayo Clinic College of Medicine and Science, 200 First Street SW, Rochester, MN 55905, USA; ^2^Center for Palliative Medicine, Department of Medicine, Mayo Clinic College of Medicine and Science, 200 First Street SW, Rochester, MN 55905, USA

## Abstract

Pain is one of the most feared symptoms that concern cancer patients and their families. Despite well-established guidelines set forth by the World Health Organization (WHO) on the treatment of cancer pain, nearly half of cancer patients report poorly controlled pain. One of the most serious side effects of systemic oral opioid use is neurotoxicity, which is characterized by altered mental status and systemic neurologic impairments. Treatment strategies are supportive in nature and focused on reducing or changing the offending opioid and correcting any metabolic deficiencies. Herein, we discuss a case of opioid-induced neurotoxicity treated with intrathecal targeted drug delivery (TDD). The timing and implementation of advanced therapies such as intrathecal TDD is not well delineated. More importantly, patients and their oncologic providers are often unaware of this useful tool in treating challenging cancer-associated pain and significantly minimizing systemic opioid side effects. To ensure that patients have comprehensive oncologic care, best-practice guidelines suggest involvement of an interdisciplinary team and coordinated care. Early referral to a pain and palliative specialist may allow for improved patient outcomes and removal of unnecessary barriers to optimal patient care.

## 1. Introduction

Pain is one of the most feared symptoms that concern cancer patients given its profound link to impaired quality of life, psychological distress, impaired sleep and relationships, and global functioning. Cancer-associated pain ranges widely, but is a common and prevalent theme for many in the advanced stages [1]. Traditionally, use of the World Health Organization (WHO) guidelines has outlined the most well-accepted algorithm for treatment of cancer-associated pain using a combination of adjuvant analgesics, nonsteroidal anti-inflammatory agents, and opioids. However, despite these guidelines, nearly one-half to one-third of cancer patients experience inadequate levels of analgesic control relative to reported level of pain intensity [2, 3].

Intrathecal targeted drug delivery (TDD) is a proven and accepted therapy in cancer patients for the treatment of uncontrolled pain refractory to systemic opioid regimens and intolerable side effects [1, 4–8]. Despite the known benefits of TDD in this patient population, significant barriers to early consultation and implantation remain [9]. Expert panel guidelines often refer to TDD as a rescue therapy after a failure of systemic oral options [10, 11]. Furthermore, the timing of TDD placement is influenced by a number of patient factors, including the ability to consent for a procedure, anticoagulation status, and provider awareness [12, 13]. Herein, we discuss opioid-associated neurocognitive toxicity and the role of intrathecal TDD.

## 2. Case Presentation

A 57-year-old female with widely metastatic high-grade serous adenocarcinoma of the ovary was referred to our tertiary palliative care clinic by her oncologist for management of severe cancer-related pain. Following her original diagnosis, she underwent a number of multimodal disease-directed therapies, including surgical resection and chemotherapy. She had significant ongoing mixed somatic and neuropathic pain in the left lower back as well as her pelvis, both sites of known metastatic disease. The back pain was noted to be a deep sharp pain without radiation to her extremities, located primarily around the region of her nephrostomy tube and into the abdomen. She would also have sharp lancinating neuropathic pain in her pelvis due to tumor burden with prolonged periods of sitting, walking, or activity.

Initial treatment consisted of multimodal pharmacologic therapy including short-acting and long-acting opioids with nonopioid and adjuvant neuropathic agents. Despite these therapies, she had progressive pain and was ultimately increased to an opioid combination of morphine sulfate controlled-release (120 mg by mouth every 8 hours) and morphine sulfate immediate-release (30–45 mg by mouth every 3 hours as needed). Early changes yielded acceptable analgesia. Conversations and medication adjustments continued over the phone, given the distance between the patient's home and our institution. However, over several weeks' time, pain progressed despite further opioid increases. Given concern for escalating pain and poor response to several attempts at altering her opioid therapy, it was advised that she present for a consultation in the palliative medicine clinic. Fortunately, focused discussions with the patient and her family around the patient's goals of care were addressed with her palliative medicine team at every meeting. She was very clear that her top priority in her ongoing medical care was to achieve acceptable analgesia. During the interview in the clinic, the patient noted significant pain with generalized discomfort, a sense of restlessness, and new muscle fasciculations. Her husband accompanied her and noted intermittent confusion. Her physical exam featured delirium (Confusion Assessment Method positive) with diffuse myoclonus.

Out of concern for opioid-induced neurotoxicity (OIN), the patient was admitted to the hospital for analgesic control and treatment of what was felt to be a toxic encephalopathy. Despite the known shared metabolic pathway (phase II metabolism) between morphine and hydromorphone, the patient's opioids were rotated from oral morphine to parenteral hydromorphone as the clinical picture continued to unfold. Further workup revealed an unremarkable head CT and EEG. Clinical evaluations and laboratory assessments suggested that, in the setting of her declining physical and renal function (creatinine 1.0 mg/dL with prior baseline 0.6 mg/dL and estimated glomerular filtration rate (eGFR) 55 ml/min/BSA with prior baseline >60 ml/min/BSA), the delirium could be the result of an accumulation of active neurotoxic morphine metabolites (morphine-3-glucuronide). In addition, she was dehydrated, constipated, and had not slept in several days. Unfortunately, her delirium persisted despite correction of metabolic derangements, hydration, and an aggressive bowel regimen.

Urology was consulted, and a nuclear medicine dimercaptosuccinic acid (DMSA) scan revealed minimal function of the left kidney, despite the presence of a nephrostomy tube. Subsequent vascular radiological investigations revealed tumor-induced thrombosis with surmised infarction of her left kidney. It was concluded that the altered renal function due to infarction likely resulted in an inability to adequately excrete the morphine, and the accumulation of polar morphine metabolites resulted in OIN. As exemplified in this case, even with opioid rotation, centrally active metabolites of hydromorphone (hydromorphone-3-glucuronide) theoretically exist, given that it follows a similar phase II metabolic glucuronidation pathway as morphine, although the relative potency and effect of such hydromorphone metabolites are thought to be significantly less than morphine. The patient continued to have signs of OIN and was eventually transitioned to parenteral fentanyl, given that fentanyl is a unique medication that is largely hepatically metabolized into inactive metabolites. Despite common lore that fentanyl and methadone are agents that do no produce active metabolites and therefore do not elicit OIN, case reports of synthetic opioids eliciting OIN do exist in the literature [14, 15]. Unfortunately, despite aggressive supportive measures and conversion of parenteral hydromorphone to parenteral fentanyl without reduction for cross-tolerance, she continued to have persistent pain with minimal resolution of her systemic neurotoxicity.

Given the concern for poorly controlled cancer-associated pain, the palliative care and pain medicine teams collaborated from the initial visit with concerning symptoms of OIN. At our institution, the palliative clinic shares space within the pain clinic, and the collaboration required a simple conversation followed by same day pain consultation. Initially, the shared thought was moving directly to intrathecal (IT) TDD therapy, as this had been discussed over numerous palliative care visits as a potential option with her physicians and was in line with her goals to achieve enhanced pain control with limited side effects. However, given the underlying metastatic disease process and concurrent cancer treatments, she had a metabolic coagulopathy due to nutritional and micronutrient deficits that required correction with vitamin k to assist in the reversal of her international normalized ratio (INR), thus allowing for any type of procedural intervention.

Ultimately, given the persistent multifactorial delirium and rapidly changing situation, the pain medicine and palliative care teams convened a family conference with the husband, the patients' health-care power of attorney, to again address the current situation and make recommendations that seemed consistent with the patient's predetermined goals. Given the rapid progression of her symptoms despite aggressive attempts to correct potentially remediable factors, the shared medical decision-making discussion included moving forward with TDD, given that this therapy was thought to be the best chance for meeting the patients' goals for comfort and hope to have meaningful interaction with family by reducing the burden of systemic side effects from oral or parenteral opioid therapy. The patient's advanced directive was clear that she appointed her husband to make decisions on her behalf if she was ever in a situation where she was unable to provide consent. Informed consent was obtained through her appointed surrogate decision maker and designated health-care power of attorney.

After correction of her metabolic coagulopathy yielding an INR <1.2, she proceeded with placement of an intrathecal TDD system (Medtronic SynchroMed™ II 40 mL pump and Ascenda catheter). The pump reservoir was placed in her right lower abdominal quadrant, and the catheter tip was placed at T10. Her TDD system was efficiently titrated to achieve acceptable levels of analgesia using a combination of opioid (hydromorphone 2 mg/mL) and local anesthetic (bupivacaine 10 mg/mL). The initial postoperative settings were 0.5 mg/day of hydromorphone in a simple continuous mode without bolus dosing (2.5 mg/day of bupivacaine). Within 24 hours of TDD placement, the patient experienced near complete resolution of her toxic encephalopathy and was able to engage in meaningful conversation with her family and health-care teams. There were no signs or symptoms of opioid withdrawal. In the early postoperative phase, she required additional dosing of oral hydromorphone, but was responsive to 2 mg orally every 3 hours as needed for breakthrough postsurgical pain totaling three to four doses per day. Given the robust response to TDD therapy, the patient was able to participate in goals of care discussions with her interdisciplinary palliative care team and elected to discharge closer to home under the provision of hospice services postoperative day (POD) two. Prior to leaving the hospital, the TDD rate was increased to 0.8 mg/day of hydromorphone, and she was provided with a personal therapy manager (PTM) allowing 0.1 mg/dose every six hours totaling four doses per day. The dose remained stable for seven days. As her oncologic disease continued to evolve, her TDD system was adjusted by her hospice provider to accommodate her daily use of oral opioid therapy. The pump was again adjusted on postoperative day nine, yielding a basal rate of 1.4 mg/day of hydromorphone with four PTM boluses of 0.15 mg/dose of hydromorphone every six hours. This dose remained for POD 9–13, and she passed away peacefully with acceptable analgesia at home with her family under the auspices of hospice two weeks after TDD implantation.

## 3. Discussion

As colleagues in pain and palliative medicine understand, there is a paucity of data regarding the exact timing of TDD in patients with advanced cancer. Despite well-validated World Health Organization (WHO) guidelines on cancer pain management that serve approximately 80% of the cancer pain population, the remaining 20% may continue to have poorly controlled pain and be at an increased risk for systemic opioid side effects [16]. Management of cancer pain is unique to the individual, and many diverse clinical scenarios are considered, ranging from chronic cancer treatment-related pain to confronting end-of-life concerns. In our practice, we strongly advocate an interdisciplinary approach, early consultation for consideration of TDD, and finally establishment of a coordinated care plan.

First, best practices to enhance patient care involve interdisciplinary teams and coordinated care [17]. True interdisciplinary care involves health-care professionals (i.e., physicians, therapists, social workers, advanced practice clinicians, chaplains, and nurses) with unique skills, expertise, and knowledge from diverse backgrounds focused on collaborating to achieve a common goal of comprehensive patient care. Integrated cancer centers recognize that comprehensive cancer care focuses on the entire individual, and consultation with oncology, radiation oncology, surgical oncology, palliative care, and pain medicine colleagues is crucial. Studies have suggested that co-consulting services that work together on managing complex cancer-associated symptoms, such as pain, often lead to enhanced analgesia, improved quality of life, and potentially reduced morbidity [18–20]. We recognize that there exist barriers in practice to comanagement, including patient-physician relationships, varying therapies offered, and patients' fear of abandonment. However, in a coordinated fashion, a comprehensive interdisciplinary approach can be taken to ensure all aspects of pain (i.e., psychological, emotional, physical, and spiritual) are being addressed in a multimodal fashion.

Secondly, building a team-based care model allows each specialist the opportunity to highlight their expertise and discuss the role of potential treatment options early in the care paradigm. Early discussion of all treatment options facilitates an environment of trust and allows the members of the patient's care team the greatest opportunity to offer interventions when most appropriate [21]. Early discussion of various therapies also often yields greater insight from the patient and their family unit in directing the course of clinical care. In our case, the patient and her husband were able to clearly articulate that they had concerns about her pain regimen and its effects from the initial visit. This prompted consultation with colleagues in interventional pain medicine to discuss the role of TDD in advanced cancer. In our experience, regular weekly meetings between interested palliative and pain providers occur to ensure discussion of patients in need of advanced pain therapies, and our patients report the discussion regarding advanced therapies early in their course of treatment as reassuring and encouraging. Initiating IT therapy early in the care paradigm allows for customization of medication dose and titration which is tailored to the individual as the disease progresses. As with any medical therapy, discussion about the risk, benefit, and alternative profile of intrathecal TDD is important for optimal outcomes. Common side effects discussed with intrathecal opioid therapies include pruritus, constipation, urinary retention, and nausea. These are typically self-limited on the order of days to weeks. More serious complications of intrathecal opioids include respiratory depression or opioid withdrawal, but these are rare and can manifest at any point during therapy. These entities are treated with either opioid reversal agents (i.e., naloxone) or opioids combined with nonopioid adjuvants (i.e., clonidine), respectively. The most serious and least common complications discussed included surgical site infection, neuraxial bleeding, and the development of a granuloma in the spinal canal which can precipitate paralysis, the need for surgical intervention, and possibly death. Despite the minimally invasive nature of the intervention, periprocedural planning is crucial to minimize potential adverse events and maximize optimal outcomes. Concerning features such as coagulopathy, encephalopathy, and neurologic deficits can be documented and potentially corrected prior to neuraxial intervention. The collaboration of the palliative care and pain programs has produced greater understanding of the role of advanced interventions and understanding of the guidelines for periprocedural considerations when providing interventions about the neuraxis [22].

Lastly, timely intervention allows for proper postimplant coordination, education, and collaboration required for ongoing care with palliative medicine and hospice providers. Effective intrathecal drug therapy management is required after implantation to ensure that the therapy is providing optimal outcomes through confirmation of the program performance, system integrity, and reports on battery longevity (on average, seven years) [12, 23, 24]. The benefits of TDD provide the opportunity to correct potentially remediable factors, such as systemic opioid-induced neurotoxicity, and treat the underlying cause of suffering and potentially extend life [10, 24–26]. All expert guidelines advocate for earlier consultation and consideration of intervention with TDD in cancer pain, particularly for medically challenging cases at risk for systemic opioid toxicity affecting pain, daily activities, quality of life, and neurocognitive function as outcomes have demonstrated reduced systemic opioid toxicity, improved pain control, and risk mitigation of oral opioid therapies [17, 23, 27].

Several randomized control trials have observed the outcomes of IDDS with comprehensive medical management (CMM) compared to CMM alone [25, 26]. Opioid side effects are feared by both patients and their physicians and more importantly are a frequent contributor to the failure of cancer pain therapy. As one may expect, the use of TDD therapy exemplified improvement in VAS pain scores, perception of pain control, and significantly reduced opioid-related toxicities. Analysis in these studies even suggests improved survival in patients with refractory cancer pain. It is important to consider that the patients who received TDD therapy had improved survival at 6 months when compared to those in the non-TDD group, and this trend held true for those who failed CMM and ended up receiving TDD therapy [25, 26].

Current guidelines suggest pursuing maximal medical therapy prior to considering implantation of a TDD [12, 23, 27]. In noncancer pain patients, an injection of opioid into the intrathecal space is initially tried with the primary goal being to identify those patients that would likely benefit from intrathecal therapy. An existing best-practice literature in cancer pain patients with a life-limiting illness does not clearly define or recommend trialing with either a single shot or continuous intrathecal catheter of intrathecal opioid therapy [23, 27]. We recommend that if dose escalation of opioid therapies results in temporary relief or unique synthetic opioids such as high-dose fentanyl or methadone is being considered, then targeted IDD should also be considered.

Originally, one of the most compelling arguments supporting the use of TDD in cancer pain focused on the cost-effectiveness of the intervention with the expectation for the patient to live at least six months. This rationale was based on data that suggested an arbitrary number, for which the intrathecal device would breakeven in terms of cost and outcome [10, 27, 28]. More recent studies have suggested that this therapy be offered to any cancer patient with a life-limiting illness suffering from intractable pain, particularly if life expectancy is thought to be approximately 3 months or more as this can significantly impact the overall pain, suffering, quality, and potentially quantity of life [23, 25–27]. Ultimately, early assessment and intervention using TDD is crucial to enhance analgesic efforts in patients with advanced cancer and represents a key option in the provision of high-quality, patient-centered supportive cancer care [16].

The process of TDD implant entails review of the patient's medical condition and comorbidities, advanced imaging of the neuraxial space (e.g., cervical, thoracic, and/or lumbar spine), and assessment of current medication regimen [17, 23]. If a patient is felt to be a good candidate by the implanting physician, a small flexible catheter is placed via a needle into the intrathecal space from a small incision in the back. This flexible catheter is then tunneled under the skin and connected to a programmable drug infusion system (about the size of a hockey puck) that is placed under the skin. Once connected and turned on, the battery-powered pump uses a small rotor to precisely propel the programmed medication dose through the catheter into the spinal canal. The total daily dose of medication is controlled by the clinician to deliver a specific dose of medication per day. Patients can utilize a personal therapy manager (PTM) to have clinician prescribed bolus doses available for breakthrough or incident-related pain flares during the day [24].

One of the most important questions implanting physicians face is the conversion of systemic opioid dose to a safe and effective intrathecal opioid dose. The application of an equianalgesic conversion ratio for oral to intrathecal opioid depends on the hydrophilic nature of the opioid medication. The only three FDA approved medicines to be used intrathecally through a TDD device are morphine, baclofen, and ziconotide. For purposes of this conversation, we will only discuss the conversion of morphine. It is well accepted that oral to parenteral morphine is a conversion factor of three, parenteral to epidural morphine is a conversion factor of ten, and epidural to IT morphine is a conversion factor of ten [29, 30]. Therefore, when calculating an “equianalgesic dose” of oral morphine to IT morphine, it roughly equates to 1 (oral): 3 (parenteral): 10 (epidural): 10 (intrathecal) or 300 times greater. In this case, at the time of TDD implantation, our patient was taking approximately 800 mg of oral morphine equivalents per day (the patient's 24-hour parenteral fentanyl use was approaching 3000 micrograms/day). Thus, when converting to an IT dose, 800 mg OME/300 = 2.67 mg/day of IT morphine. By convention, assuming a conversion ratio of 5 : 1 when converting from morphine to hydromorphone (typical conversion range 4–7 mg morphine:1 mg hydromorphone), 2.67 mg of IT morphine/5 = 0.5 mg of IT hydromorphone. Thus, the pump was programmed to administer a total daily dose of 0.5 mg of IT hydromorphone as the primary medication. It was elected to not dose reduce in this situation for several reasons, including the patient's significant opioid tolerance, her poor prognosis, and multiple failed attempts to treat her refractory cancer pain.

The vast majority of interventional pain practices follow the updated 2017 Polyanalgesic Consensus Conference (PACC) guidelines when implanting and managing TDD systems for cancer pain. First-line medication selection includes FDA approved medications, including ziconotide and morphine. Additionally, fentanyl and hydromorphone are both considered first line in treating cancer pain particularly when combined with nonopioid adjuvants such as bupivacaine [23]. The addition of adjunct medications such as bupivacaine is useful to alter sensory processing while sparing motor function at low doses and offers potent synergistic effects when paired with IT opioid therapy. Typical range for IT bupivacaine is very dependent on the patient, but data would suggest that modest analgesic effect occurs around 8–12 mg/day [23]. Certainly, a minor surgical procedure such as TDD is not without risk. The primary adverse events experienced with neuraxial analgesia are related to nausea, pruritus, postdural puncture headache, urinary retention, endocrine dysfunction including hypogonadism (particularly with longer term therapy), bleeding around the neuraxis, infection of the neuraxis, and respiratory depression. We counsel all patients on these potential side effects, which occur rarely [12].

The goal of cancer-directed pain care should focus on reducing delay and apprehension regarding IT TDD intervention, thus reducing the known morbidity and mortality associated with oral systemic opioid therapies. We strongly encourage collaborative communication in patients that are terminally ill, as the available resources of hospice programs vary greatly and may impact the medical decision-making around the application of TDD therapy near the end of life [27]. In our experience, commercial vendors are very much willing to ensure that patients and their health-care teams have the knowledge and support needed to effectively and safely manage the TDD after implant, including support of hospice agency staff training/education for medication refills and adjustments. As the oncologic disease often changes over time, the use of targeted drug delivery is meant to change with the patient and can be adjusted with relative simplicity. In this case, the patient had weekly adjustments over the ensuing weeks after enrollment in hospice and ultimately ended with hydromorphone totaling 2.0 mg/day, with bupivacaine totaling 10 mg per day. Each adjustment of the intrathecal system attempted to account for the dynamic pain symptoms as well as the daily alteration of oral opioid therapies and course of disease trajectory.

## 4. Summary

In summary, cancer-associated pain continues to be a prevalent concern for numerous patients, their families, and their treating providers. The burden of uncontrolled pain symptoms can greatly affect function, daily activities, interpersonal relationships, and quality of life at the end of life. Comprehensive cancer care includes a multidisciplinary team to ensure that clear communication and consultation with collaborating providers guarantees the best possible outcome for each patient. As highlighted in this case, consideration of an intrathecal TDD system is an important therapeutic intervention that may correct a known systemic opioid side effect and allow patients' greater control during an otherwise tumultuous journey. We strongly recommend considering this option sooner rather than later in treatment of cancer pain.
